# Supplementary material for: Ablation of Acid Ceramidase Impairs Autophagy and Mitochondria Activity in Melanoma Cells
Source: Int J Mol Sci. 2021 Mar 23;22(6):3247. doi: 10.3390/ijms22063247 (PMC8004726; doi:10.3390/ijms22063247)
Supplement: Supplementary file 1 [file ijms-22-03247-s001.pdf]

Supplementary Information

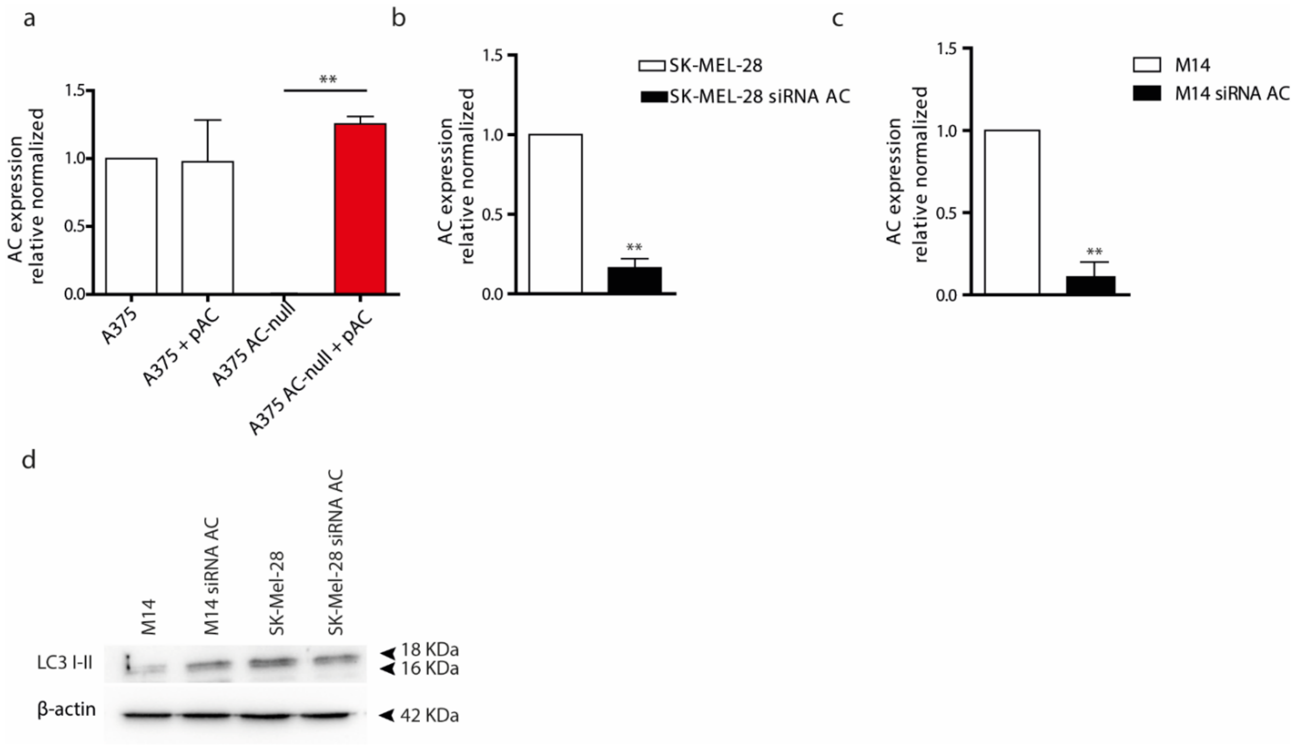

Supplementary Figure S1. (**a**) qRT-PCR of AC expression in A375 and A375 AC-null cells transfected or not with pAC. Results are expressed as mean ± SD, with each experiment performed with three biological and three technical replicates (** $p < 0.01$. One Way ANOVA and Bonferroni post hoc test). (**b,c**) M14 and SK-Mel-28 cells were transfected or not with AC siRNA. After 48 h, qRT-PCR of AC was performed. Results are expressed as mean ± SD, with each experiment performed in three biological and technical replicates (** $p < 0.01$ Student *t*-test). (**d**) Western blot on LC3 I-II on M14 and SK-Mel-28 transfected or not with AC siRNA for 48h. Lipidomic profiles of A375 and A375 AC-null cells are available in a previous work published by our team [16].
